# The Nuclear Matrix Protein Megator Regulates Stem Cell Asymmetric Division through the Mitotic Checkpoint Complex in *Drosophila* Testes

**DOI:** 10.1371/journal.pgen.1005750

**Published:** 2015-12-29

**Authors:** Ying Liu, Shree Ram Singh, Xiankun Zeng, Jiangsha Zhao, Steven X. Hou

**Affiliations:** The Basic Research Laboratory, National Cancer Institute, National Institutes of Health, Frederick, Maryland, United States of America; University of Melbourne, AUSTRALIA

## Abstract

In adult *Drosophila* testis, asymmetric division of germline stem cells (GSCs) is specified by an oriented spindle and cortically localized adenomatous coli tumor suppressor homolog 2 (Apc2). However, the molecular mechanism underlying these events remains unclear. Here we identified Megator (Mtor), a nuclear matrix protein, which regulates GSC maintenance and asymmetric division through the spindle assembly checkpoint (SAC) complex. Loss of Mtor function results in Apc2 mis-localization, incorrect centrosome orientation, defective mitotic spindle formation, and abnormal chromosome segregation that lead to the eventual GSC loss. Expression of mitotic arrest-deficient-2 (Mad2) and monopolar spindle 1 (Mps1) of the SAC complex effectively rescued the GSC loss phenotype associated with loss of Mtor function. Collectively our results define a new role of the nuclear matrix-SAC axis in regulating stem cell maintenance and asymmetric division.

## Author Summary

Like many stem cells, the adult *Drosophila* male GSC often divides asymmetrically to produce one new stem cell and one gonialblast. The asymmetric division of GSC is specified by perpendicular orientation of the mitotic spindle to the hub-GSC interface and localization of Apc2. Here we show that Tpr/Mtor regulates GSC self-renewal and asymmetric division through the SAC complex. We found that Mtor cell-autonomously required in both GSCs and CySCs to regulate their self-renewal. Loss of Mtor function affects expression and localization of Apc2 and E-cadherin. We further found that Mtor is required for the correct centrosome orientation, mitotic spindle formation, and chromosome segregation. These defects are rescued by SAC complex components, Mps1 and Mad2. These data together suggest that Mtor regulates GSC asymmetric division and maintenance through the mitotic spindle checkpoint complex. We suggest that disruption of the Tpr-SAC pathway might lead to chromosome instability, chromosome lagging, and aneuploidy, stem cell division defects, and thereby tumor development.

## Introduction

**Fig 1:**
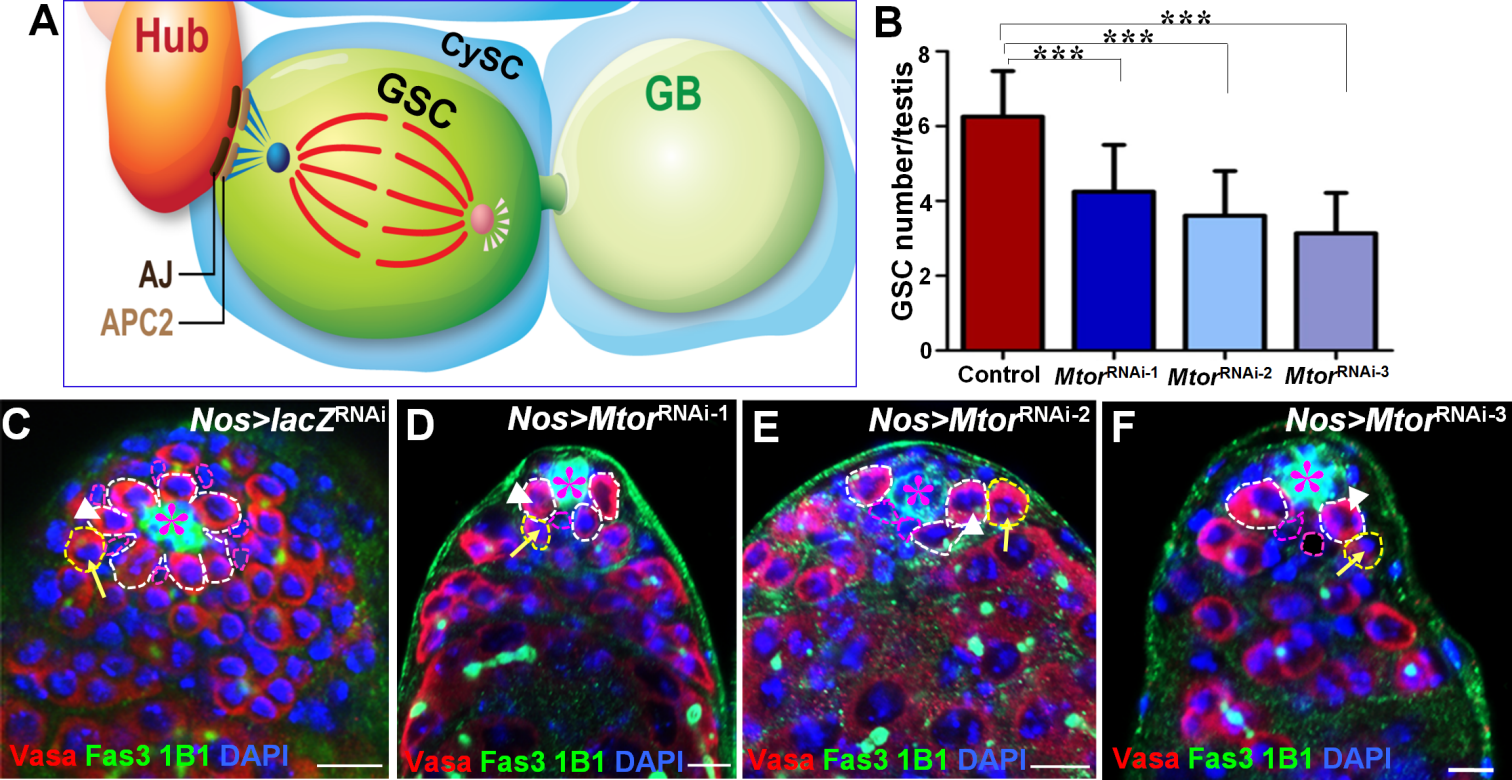
Mtor regulates GSC maintenance. (A) A schematic drawing of the asymmetric division of a *Drosophila* male GSC. Both GSCs (green) and CySCs (blue) are anchored around the hub (orange) through adherens junctions (brown). Asymmetric division of a GSC results in a new GSC (green) and a gonialblast (GB) (light green). In the GSC (green), the mother centrosome (dark blue) nucleates more microtubules (dark blue) than the daughter centrosome (pink), which may help to asymmetrically deliver Apc2 (beige) to the cortex, where GSCs contact the hub. At the cortex, Apc2 concentrates at the cell–cell junction (brown) to anchor the mitotic spindles from the mother centrosome perpendicular to the hub, but the daughter centrosome remains on the opposite side (pink). As a result, GSCs divide asymmetrically. (B) Quantification of the number of GSCs associated with the hub (7 days old flies were dissected and stained for each genotype). *Nos-Gal4*-driven *Mtor* ^RNAi-1^ (n = 35), *Mtor* ^RNAi-2^ (n = 39), or *Mtor* ^RNAi-3^ (n = 41) caused a significant decrease in the number of GSCs associated with the hub compared to the control *Nos*-*Gal4*-driven *LacZ* ^RNAi^ (n = 27). ***p<0.0001. Error bars represent SD. (C-F) GSCs in testes of wild-type control (*Nos>lacZ* ^RNAi^) (C), *Nos>Mtor* ^RNAi-1^ (D), *Nos>Mtor* ^RNAi-2^ (E), and *Nos>Mtor* ^RNAi-3^ flies (F) were examined by staining with anti-vasa [red, marks all germ cells including GSCs in white dotted circle (one depicted in a white arrowhead); yellow dotted circle marks the GB (one depicted in yellow arrow)], anti-Fas3 (green, marks the hub cells, asterisks), anti-1B1 (green in dot and branched marks the spectrosomes and fusomes respectively), and DAPI (blue). Some CySCs positioned adjacent to the hub cells (pink dotted circle) after some GSCs were depleted in *Nos>Mtor* ^RNAi^ testes (D-F). All flies were cultured for 7 days at 29°C before dissection. Scale bars represent 10 μm.

Germline stem cells (GSCs) from the *Drosophila* testis provide one of the best genetic systems to study stem cell regulation. At the tip of the *Drosophila* testis (apex) is a germinal proliferation center, which contains the germline and somatic stem cells that maintain spermatogenesis (Fig 1A) [1–5]. Each GSC is encysted by two somatic cyst stem cells (CySCs). Both GSCs and CySCs anchor to a group of 12 nondividing somatic cells, called the “hub”, through cell-adhesion molecules [6–9]. The hub defines the stem-cell niche by expressing the growth factor Unpaired (Upd), the ligand that activates the Janus kinase–signal transducer and activator of transcription (JAK-STAT) pathway in adjacent GSCs and CySCs to regulate their self-renewal [10,11]. In addition, hedgehog (Hh) [12–14], and bone morphogenetic protein (BMP) [9,15] signaling also play important role in GSC and CySC maintenance. During GSC division, the mother (old) centrosome remains anchored near the niche, while the daughter centrosome migrates to the opposite side of the cell, thereby assembling a mitotic spindle perpendicular to the hub [16–18]. In addition, the mother centriole nucleates more microtubules than the daughter centriole, which may help in asymmetric delivery of Apc2 to the cortex where GCSs contact the hub [18]. At the cortex, Apc2 and E-cadherin together anchor the spindles of mitotic GSCs perpendicular to the hub [19]. Therefore, only one daughter cell will contact the hub and receive JAK-STAT signaling to maintain stem cell identity, while the daughter cell at the other end of the mitotic spindle will experience a weaker signal and initiate differentiation. However, it is not known what molecular mechanism regulates asymmetric Apc2 localization at the niche-GSC interface.

To identify new regulators of stem cell asymmetric division in the *Drosophila* testis, we carried out a screen in which a collection of transgenic RNAi lines [20–22] were crossed with *act-Gal4; tub-Gal80* ^*ts*^ flies (referred to as *Act* ^*ts*^). The adult flies were shifted to the restrictive temperature (29°C-to inhibit Gal80 activity and induce Gal4-activity) from 18°C (Gal80 is active) and cultured at different time intervals. The flies were then dissected, stained, and examined for GSCs phenotype under confocal microscopy. One of the first few genes identified in this screen was *Mtor*. *Mtor* knockdown by transgenic RNAi resulted in a significant decrease of GSCs in the testes compared to the wild-type flies. Mtor belongs to a conserved family of coiled-coil proteins [translocated promoter region (Tpr) in vertebrates and myosin-like proteins 1 and 2 (Mlp1/2) in yeast] [23–25] that make up nuclear basket of the nuclear pore complex in vertebrates [26–29], and nuclear matrix in flies [27]. Tpr/Mtor plays an important role in regulating the SAC. The SAC delays anaphase until chromosomes are bioriented on the mitotic spindle. Recent studies demonstrated that Tpr/Mtor regulates the SAC by either controlling the kinetochore localization of Mad1 and Mad2 [26–27, 29–30] or by targeting Mad1 to the nuclear pore to direct mitotic checkpoint complex (MCC) assembly during interphase [28].

In this study, we found that loss of *Mtor* function affects GSC maintenance and asymmetric division. Knockdown *Mtor* results in Apc2 mis-localization, defects of mitotic spindle formation and chromosome segregation, and eventual GSC loss. Expression of Mad2 and Mps1 of the SAC complex effectively rescued the GSC loss phenotype associated with loss of *Mtor* function. Our results suggest that a nuclear matrix-SAC axis regulates GSC maintenance and asymmetric division through the Mtor-Mps1/Mad2 pathway.

## Results

### Mtor is required in both GSCs and CySCs

As described above, we identified *Mtor* in a genetic screen for new regulators of stem cell fates in the *Drosophila* testis. *Mtor* knockdown by transgenic RNAi resulted in a significant decrease of GSCs in the testes compared to the wild-type testes. To further understand the function of Mtor in the germline or in the soma, we knocked down *Mtor* by using cell-type–specific Gal4s. We used three independently generated *UAS-Mtor* ^RNAi^ transgenic fly lines. We found that depleting *Mtor* in the germ cell lineage using *Nanos* (*Nos)-Gal4* resulted in a significant decrease in the number of GSCs associated with the hub compared to the wild-type control (Figs 1B–1F and S3A and S3B).

We also knocked down *Mtor* in the CySC lineage using the *c587-Gal4* driver in combination with a temperature-sensitive Gal80 inhibitor [31]. Depleting *Mtor* in the CySC lineage (*c587* ^*ts*^ *>Mtor* ^RNAi^) caused defects in GSC differentiation (S1B and S1F and S1H Fig) similar to those seen in cyst cells defective in the epidermal growth factor receptor (EGFR) signaling pathway [32–36; S1C and S1D Fig]. We further examined CySC changes in *c587* ^*ts*^ *>Mtor* ^RNAi^ flies using Traffic Jam (Tj) (a transcription factor expressed in CySCs, early cyst cells, and the hub cells) and Eyes absent (Eya) (a transcription factor expressed in early cyst cells) markers (S1E–S1H Fig). In comparison with those in the wild-type testes, the Tj-positive (S1 Fig, compare S1E and S1F Fig) and Eya-positive cells (S1 Fig, compare S1G and S1H Fig) were pushed away from the niche by the expanding undifferentiated germ cells in the testes of *c587* ^*ts*^ *>Mtor* ^RNAi^ flies, suggesting that the *Mtor*-deficient CySCs have disadvantage in niche occupancy.

We further knocked down *Mtor* in the hub cells using *unpaired (upd)-Gal4* (S2B Fig), in adult fly posterior midgut intestinal stem cells (ISCs) and progenitors using *escargot (esg)-Gal4* in combination with a temperature-sensitive Gal80 inhibitor [31] (*esg* ^ts^ *>Mtor* ^RNAi^) (S2D Fig), and in nine other types of cells using the cell-type–specific Gal4s (S2E Fig). Knockdowns of *Mtor* in the hub cells, ISCs and progenitors, and eight other cell types [lymph gland (plasmatocytes and crystal cells, *collagen (Cg)-Gal4)* [37], *Drosophila insulin like peptide 2 (Dilp2)-Gal4* [38], leading edge *(LE*-*Gal4)* [39], fat body (*pumpless (ppl)-Gal4)* [40], hindgut (*brachyenteron (byn)-Gal4)* [41], hemocytes (*serpent* ^*hemo*^ *(srp* ^*hemo*^ *)-Gal4)* [42], corpus allatum (*Aug21-Gal4)* [43], and hemocytes (*hemolectin (hml)-Gal4)* [44]], have no obvious phenotypes compared to those cells in the wild-type flies. Only Knockdowns of *Mtor* using wing-specific *(MS1096)-Gal4* [45] **(** *ms1096>Mtor* ^RNAi-2^) resulted in wing phenotype (S2E Fig). Further, we found that knock down of *Mtor* in adult ISCs has no effect on spindle orientation and chromosome segregation (S6E and S6F Fig). These data suggest that Mtor functions specifically in GSC of *Drosophila*.

Using antibodies to Mtor [46], we detected Mtor in both the germline and soma in the wild-type (S3C Fig), but not in the Mtor-depleted testis (*Act* ^ts^ *>Mtor* ^RNAi^) (S3D Fig), suggesting that the RNAi almost completely depleted Mtor protein expression. Further, we found that Mtor expresses at the nuclear pores, which is confirmed by co-staining the Mtor with nucleoporin 98 (NUP98) [32] (S3E and S3F Fig).

### Mtor cell-autonomously regulates maintenance of GSCs

**Fig 2:**
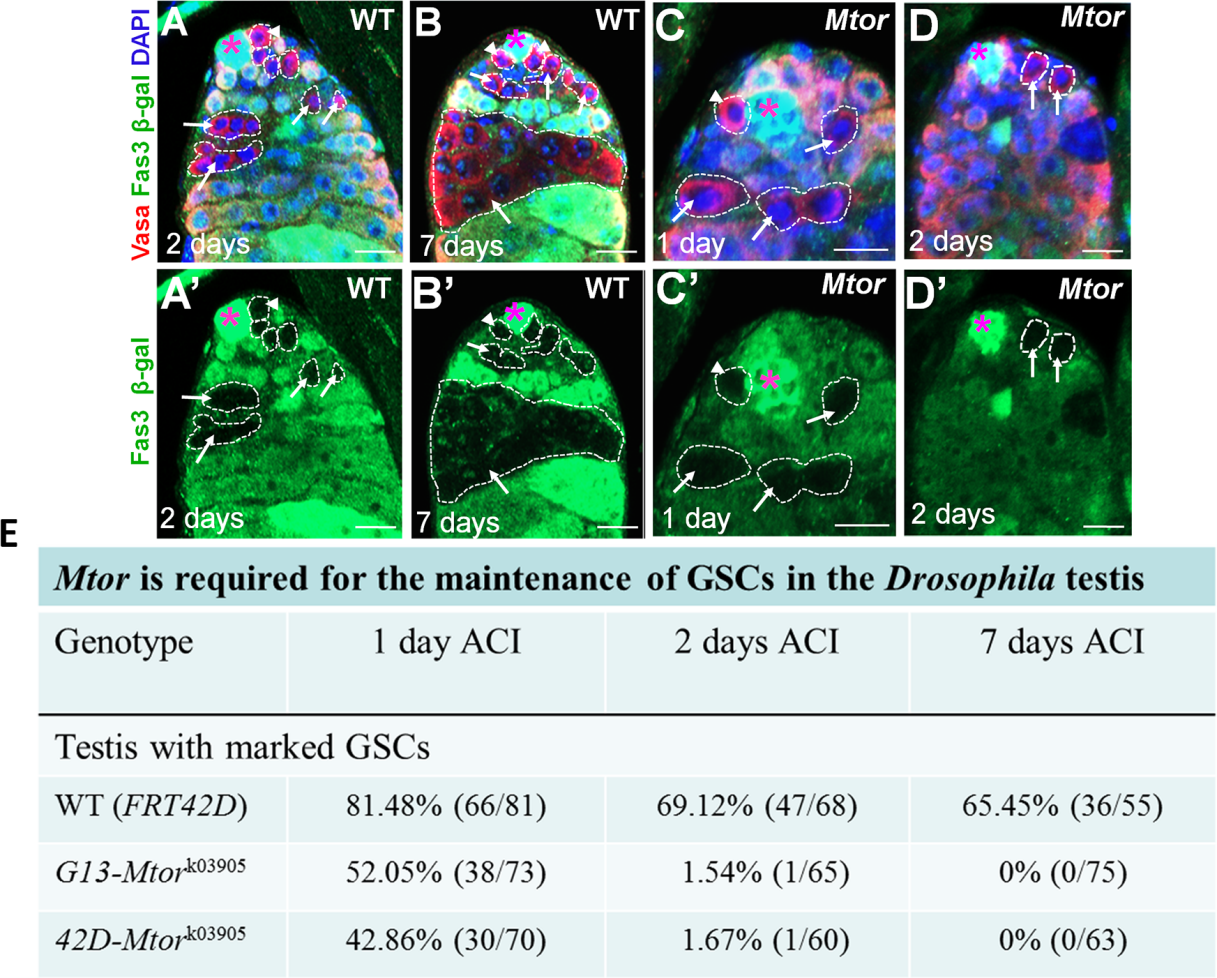
*Mtor* cell-autonomously regulates GSC maintenance. (A–D’) are confocal sections through the apex of the testes containing wild-type (A–B’) or *Mtor* ^k03905^ (C–D’) clones at the indicated times ACI. The testes were immunostained with anti-vasa (red, marks all germ cells including GSCs), and Fas3 (green, hub cells-asterisks). The wild-type or *Mtor* ^k03905^ GSC clones are β-galactosidase (green) negative. Dotted lines in A’, B’, C’, D’ represent GSC clones (arrowhead) and their differentiating progeny (arrow). Scale bars represent 10 μm. (E) Quantitative data of β-galactosidase–negative clones in wild-type control (*FRT* ^42D^ *-piM*), *FRT* ^G13^ *-Mtor* ^k03905^, or *FRT* ^42D^ *-Mtor* ^k03905^ fly testes at 1 and 2 days ACI.

To further examine the function of Mtor in GSCs, we generated negatively marked GSC clones of wild-type or *Mtor* ^k03905^ [46] flies using the FLP/FRT mosaic analysis technique [47]. Testes with LacZ (*arm-lacZ*)-negative clones were counted 1, 2, and 7 days after clone induction (ACI). As expected, in *FRT* ^42D^ control testes, we were able to find many LacZ-negative GSCs and their differentiated progenies (Fig 2A–2B’ and 2E) at 1, 2, and 7 days ACI. At 1 day ACI, we were also able to find LacZ-negative GSCs and their differentiated progenies (Fig 2C, 2C’ and 2E) in *FRT* ^42D^-*Mtor* ^k03905^ and *FRT* ^G13^-*Mtor* ^k03905^ testes. At 2 days ACI, LacZ-negative *Mtor* homozygous mutant GSCs were recovered at negligible levels compared to control clones (Fig 2D, 2D’ and 2E). At 7 days ACI, we were unable to find a single LacZ-negative GSC or differentiated germ cell in *Mtor-*mutant testes (Fig 2E).

We examined cell death using anti-caspase 3 (Cas3) staining and found a significant increase in dead cells in the testes of *FRT* ^42D^ *-Mtor* ^k03905^ mosaic clones at 1 day ACI (S4B Fig) compared to wild-type control flies (*Nos>LacZ* ^RNAi^) (S4A Fig). However, the dead cells were outside the GSC zone. Consistent with this observation, coexpression of the pan-caspase inhibitor p35 did not suppress the GSC loss phenotypes in the *Mtor-*deficient testes (S4C and S4D Fig), indicating that the GSC loss in the *Mtor-*deficient testes maybe not through apoptosis.

### Mtor cell-autonomously regulates maintenance of CySCs

**Fig 3:**
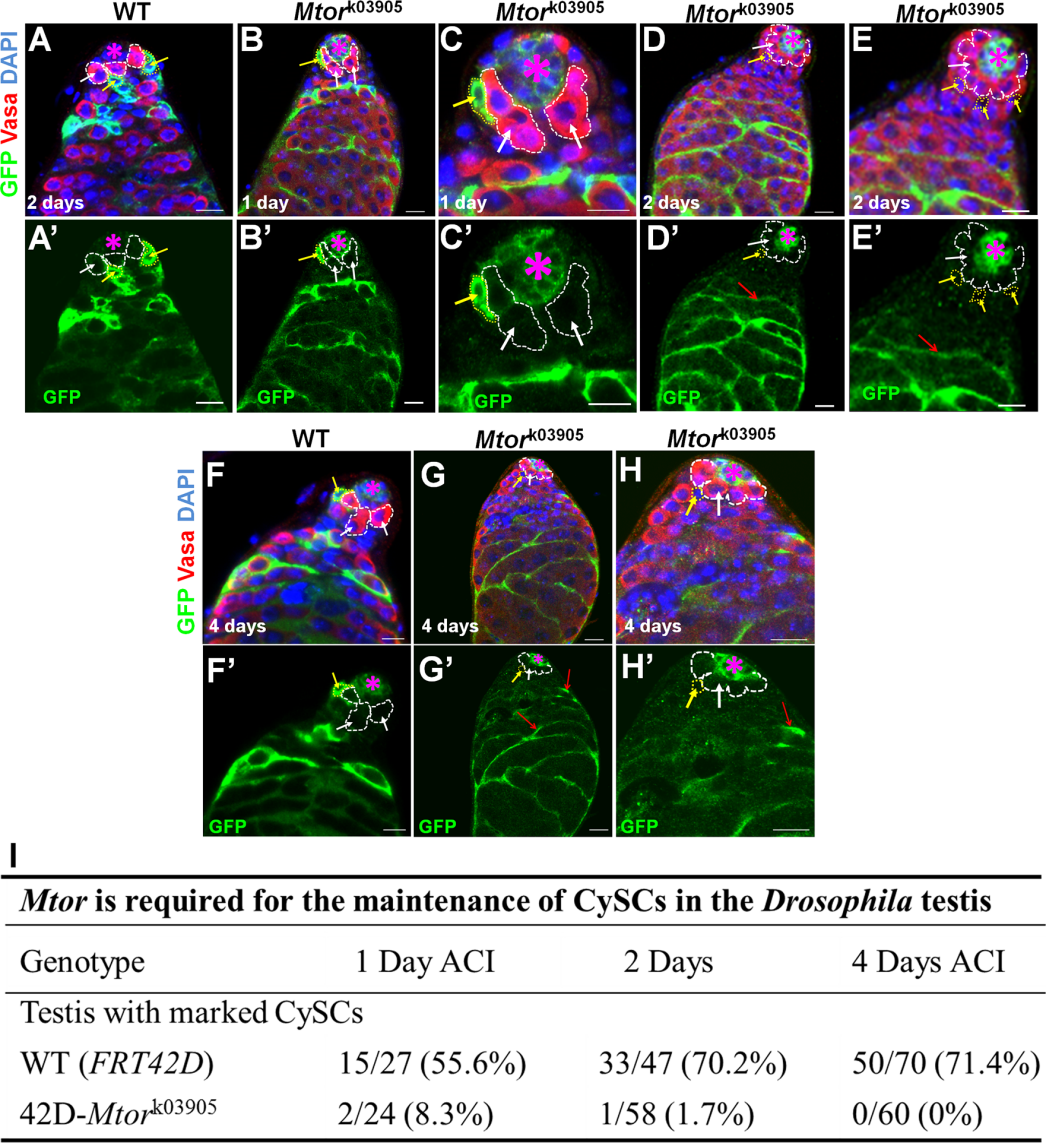
*Mtor* cell-autonomously regulates CySC differentiation. GFP^+^ clones were generated in the testes of wild-type control (*FRT* ^42D^ *-piM*; A-A’ and F-F’) or FRT^42D^ *-Mtor* ^k03905^ (B-E’ and G-H’) flies using the MARCM technique and stained at 1, 2, and 4 days ACI with the anti-GFP (green), anti-Vasa (red) and DAPI (blue). In FRT^42D^ control testes, we were able to find many GFP-positive CySCs and their differentiated progenies (A-A’, F-F’, I) at 1, 2, and 4 days ACI. In *FRT* ^42D^ *-Mtor* ^k03905^ testes, we were rarely able to find GFP-positive CySCs at 1 and 2 days ACI (B-E’, I). At 4 days ACI, we were not able to find a single GFP-positive CySC in 60 *Mtor-*mutant testes (G-H’, I). However, we could find many GFP-positive differentiated cyst cells (B-E’ and G-H’) in *Mtor*-mutant testes at 2 and 4 days ACI. CySCs are highlighted by yellow dotted lines and yellow arrows. White dotted lines arrows highlight GSCs and red arrows point to differentiated cyst cells. Red asterisks mark hubs. Scale bars represent 10 μm. (I) Quantitative data of GFP-positive CySC clones in wild-type control (*FRT* ^42D^ *-piM*) or *FRT* ^42D^ *-Mtor* ^k03905^ fly testes at 1, 2, and 4 days ACI.

We also generated GFP positively marked CySC clones of wild-type or *Mtor* ^k03905^ flies using the mosaic analysis with a repressible cell marker (MARCM) technique [48] (Fig 3). As expected, in *FRT* ^42D^ control testes, we were able to find many GFP-positive CySCs and their differentiated progenies (Fig 3A, 3A’, 3F, 3F’ and 3I) at 1, 2, and 4 days ACI. In *FRT* ^42D^ *-Mtor* ^k03905^ testes, GFP-positive *Mtor* homozygous mutant CySCs were recovered at negligible levels compared to the control clones (Fig 3B–3E’ and 3I) at 1 and 2 days ACI. At 4 days ACI, we were unable to find a single GFP-positive CySC (Fig 3G–3H’ and 3I). However, we could find many GFP-positive differentiated cyst cells (Fig 3D’, 3E’, 3G’ and 3H’) in *Mtor*-mutant testes at 2 and 4 days ACI. These results together suggest that Mtor is required for CySC self-renewal or attachment to the niche as suggested previously [32] in the *c587* ^*ts*^ *>Mtor* ^RNAi^ flies (S1H Fig). Further, we did not detect any cell death in CySCs lineages in loss of Mtor testes.

### Loss of Mtor function affects expression and localization of Apc2 and E-cadherin

**Fig 4:**
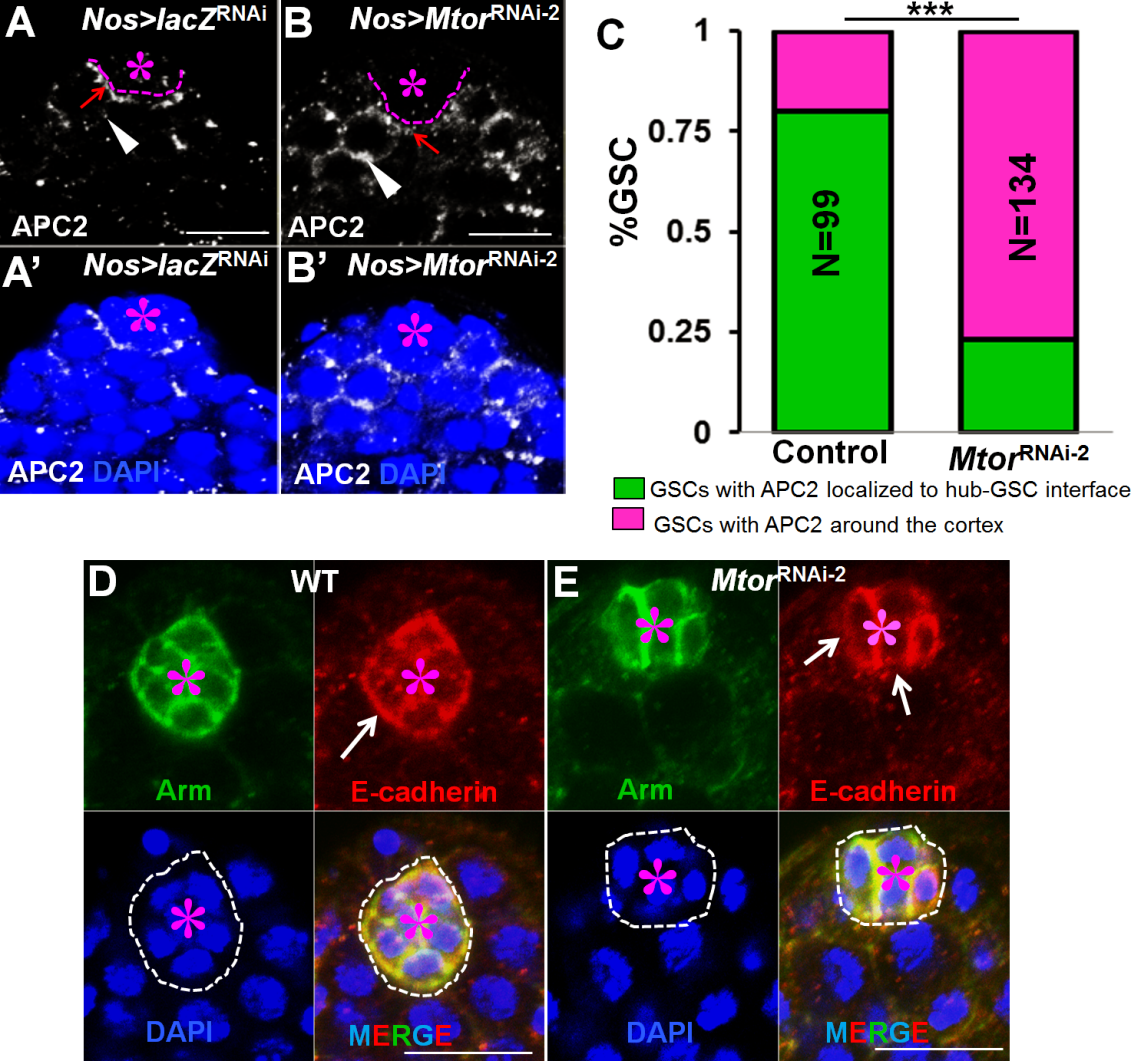
Loss of Mtor function affects expression and localization of Apc2 and E-cadherin. (A-B’) Apc2 (white) localization was examined in wild-type control (*Nos>lacZ* ^RNAi^) (A-A’) and *Nos>Mtor* ^RNAi-2^ (B-B’) testes. Red arrows point to Apc2 localization to the hub-GSC interface and white arrowheads point to Apc2 localization over the GSC cortex. The yellow stars indicate the hub. (C) Quantitation of GSCs with Apc2 localization to the hub-GSC interface versus GSCs with Apc2 localization over the GSC cortex in wild-type control (*Nos>lacZ* ^RNAi^) or *Nos>Mtor* ^RNAi-2^ flies. ***p< 0.0001, Student tests. Error bars represent SD. (D, E) E-cadherin (red) and Arm (green) localization were examined in wild-type control (*Nos>lacZ* ^RNAi^) (D) and *Nos>Mtor* ^RNAi-2^ (E) testes. White arrows point to E-cadherin localization to the hub-GSC interface. Red asterisks mark hubs. Scale bars are 10 μm in all panels. All flies were cultured for 3 days at 29°C before dissection.

During wild-type GSC division, the mother (old) centrosome remains anchored near the niche and Apc2 is localized to the interface of the niche and GSCs to anchor the spindles of mitotic GSCs perpendicular to the hub [18,19]. We examined Apc2 localization in wild-type or Mtor-depleted GSCs. After shifting the flies from 18ºC to 29ºC for 3 days, most of the Apc2 was enriched at the cortical region of GSCs adjacent to the hub-GSC interface in wild-type GSCs (Fig 4A, 4A’ and 4C), while a significant amount of Apc2 was distributed over GSC cortex from the hub-GSC interface in the GSCs remaining at the hub in Mtor-depleted (*Nos>Mtor* ^RNAi^) testes (Figs 4B, 4B’ and 4C and S4E and S4F). Similarly, we found that the expression and localization of E-cadherin at the hub-GSC interface were significantly reduced in Mtor-depleted testes in comparison with those in the wild-type testes (Fig 4D and 4E).

### Mtor function is required for the correct centrosome orientation, mitotic spindle formation, and chromosome segregation

**Fig 5:**
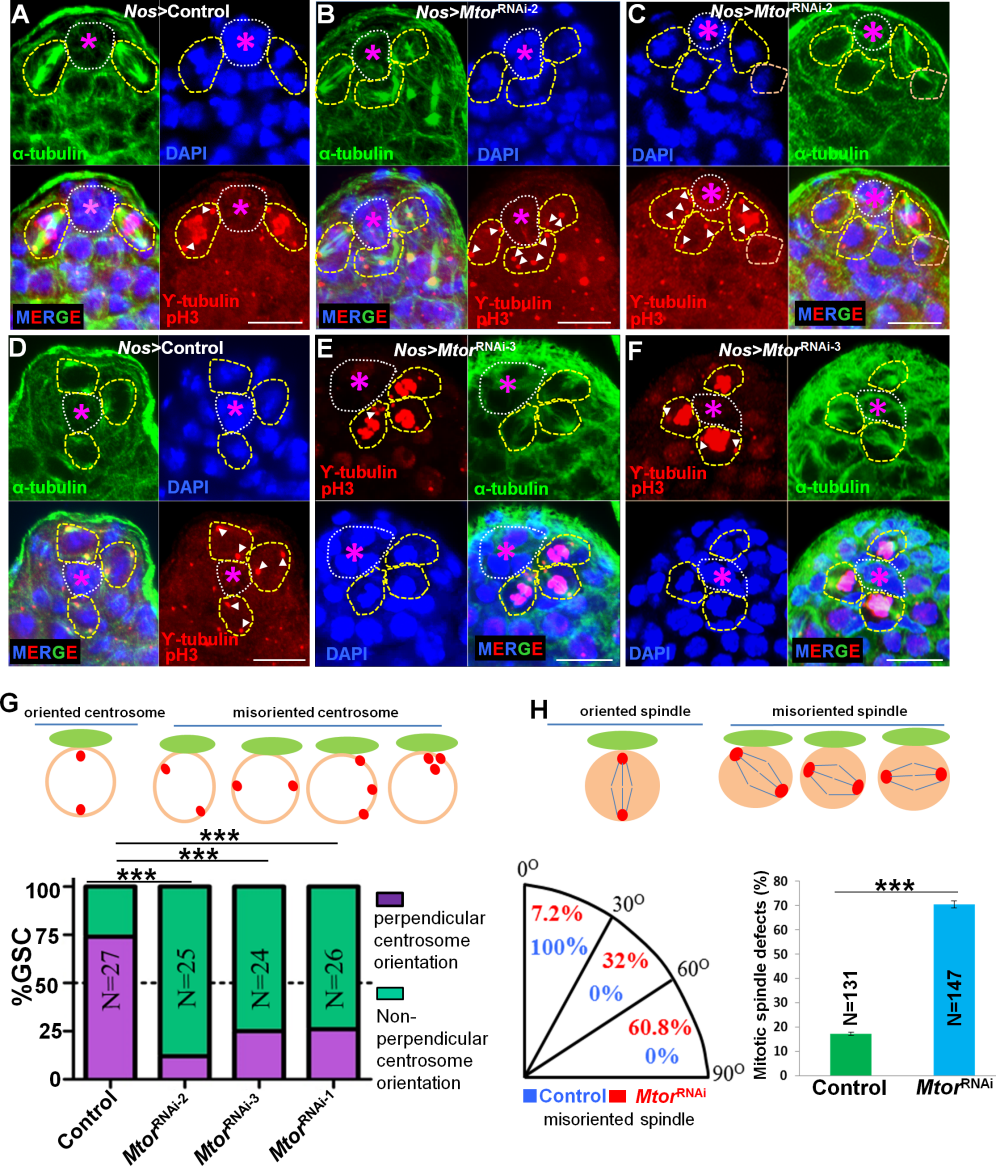
Mtor function is required for the correct centrosome orientation, mitotic spindle formation, and chromosome segregation. (A-F) Centrosome orientation and number, chromosome segregation and mitotic spindles were examined in testes of wild-type control (*Nos>lacZ* ^RNAi^) (A, D) and *Nos>Mtor* ^RNAi-2^ (B, C) and *Nos>Mtor* ^RNAi-3^ (E, F) flies. The testes were stained with α-tubulin (green), γ-tubulin, pH3 (red), and DAPI (blue). Flies were cultured for 3 days (A-D) and 7 days (E, F) at 29°C before dissection The white broken-line circles indicate the hub (asterisks). GSCs with centrosome localization and spindle orientiation are highlighted with yellow dotted lines and white arrowheads (point to centrosomes). Scale bars are 10 μm in all panels. (G) Quantitation of GSCs with oriented versus misoriented centrosomes in GSCs with two centrosomes in wild-type control (*Nos>lacZ* ^RNAi^), *Nos>Mtor* ^RNAi-1^, *Nos>Mtor* ^RNAi-2^, or *Nos>Mtor* ^RNAi-3^ flies. *** p< 0.0001, Student tests. Error bars represent SD. (H) Summarized the angles of spindle orientation and quantitation of GSCs with mitotic spindle defects in wild-type control (*Nos>lacZ* ^RNAi^) and *Nos>Mtor* ^RNAi-2^ flies. Error bars represent S.E.

We further examined centrosome orientations in wild-type or Mtor-depleted GSCs. After shifting the *Nos>Mtor* ^RNAi^ flies from 18°C to 29°C for 3 days, we found that in the Mtor-depleted testes, remaining GSCs at the hub had an increased frequency of misoriented or multiple centrosomes (Figs 5B, 5C and 5E–5G and S5B–S5D) as compared to GSCs in the wild-type testes (Figs 5A, 5D and 5G and S5A and S6A).

Besides the Apc2 localization and centrosome orientation defects, we also observed severe microtubule spindle and chromosome segregation defects in the Mtor-depleted GSCs. After shifting the *Nos>Mtor* ^RNAi^ flies from 18°C to 29°C for 3 days, we found that many phospho-Histone H3 (pH3)-positive GSCs exhibited lagging and scattered chromosome phenotypes (S5B–S5D Fig). The phenotypes became worse after shifting the *Nos>Mtor* ^RNAi^ flies from 18°C to 29°C for 7 days (Figs 5C and 5E and S5F–S5H and S6B–S6E). Most of the pH3-positive GSCs were detached from the hub (Figs 5C and S5F–S5H and S6B–S6E). The microtubule spindles formed were incomplete, unfocused, only half, and/or without clear spindle poles (Figs 5B, 5C and 5E–5H and S5F–S5H and S6B–S6E). At anaphase, some spindles remained, bridging the separated chromosomes (S6B Fig), and the chromosomes were lagging and scattered (Figs 5E and 5H and S5F–S5H and S6E).

These observations suggest that the function of Mtor is cell-autonomously required for the correct centrosome orientation, mitotic spindle formation, chromosome segregation, and localization of Apc2 to the hub-GSC interface.

### Expression of SAC components rescued GSC defects of *Mtor* knockout

**Fig 6:**
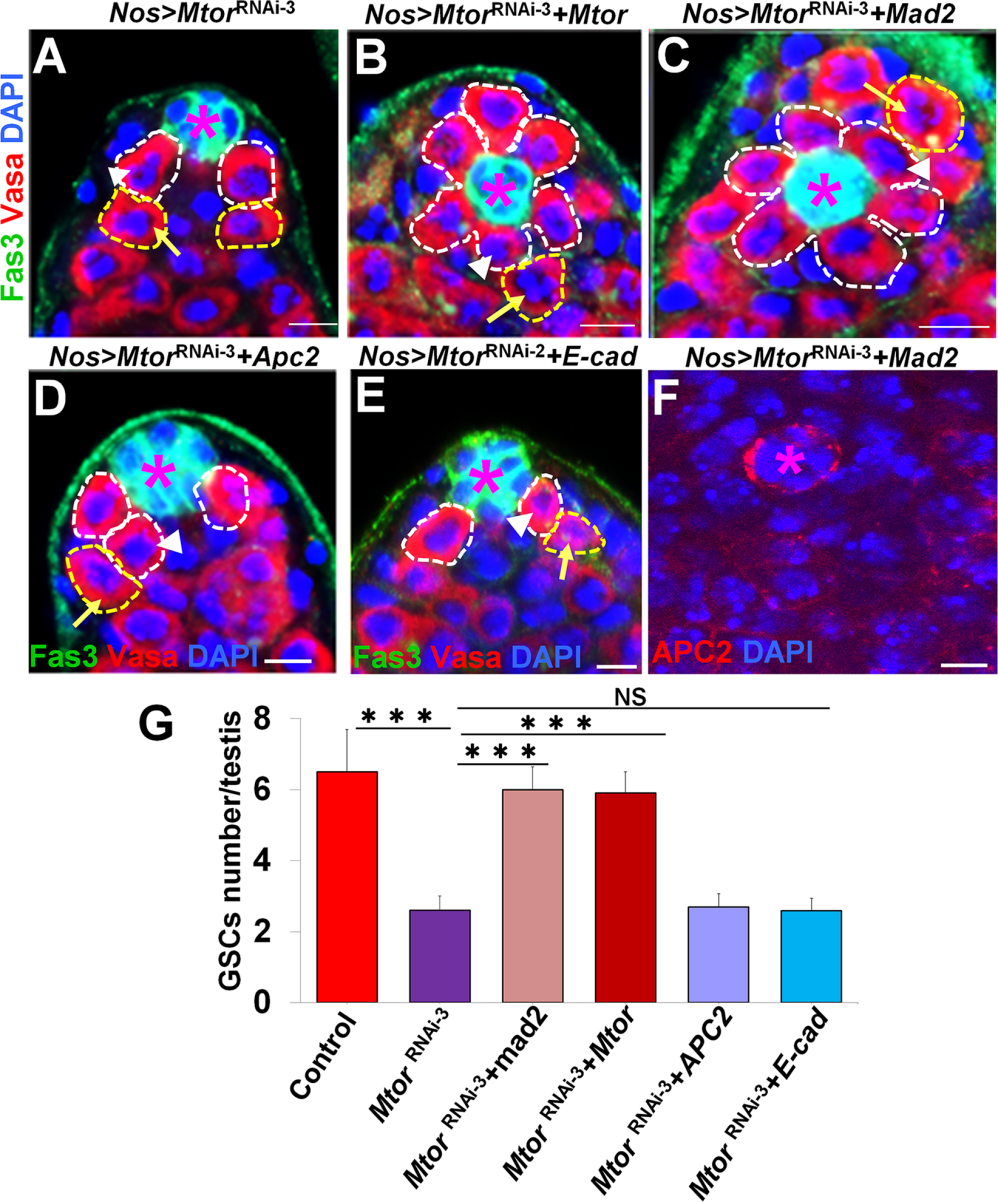
Expression of SAC components rescued GSC defects of *Mtor* knockout. (A-E) GSCs in testes of *Nos>Mtor* ^RNAi-3^ (A), *Nos>Mtor* ^RNAi-3^+*Mtor* (B), *Nos>Mtor* ^RNAi-3^+*mad2* (C), *Nos>Mtor* ^RNAi-3^+*Apc2* (D), and *Nos>Mtor* ^RNAi-2^+*E-cad* (E) flies were examined by staining with Fas3 (green, hub cells-asterisks), anti-vasa (germ cells including GSCs, white dotted circle (one depicted in a white arrowhead); yellow dotted circle marks the GB (one depicted in yellow arrow) and DAPI (blue). (F) Apc2 (red) localization was examined in *Nos>lacZ* ^RNAi^ *+Mad2* testis. Scale bars are 10 μm in all panels. (G) Quantification of the number of GSCs associated with the hub. Coexpression of *Mtor* (n = 42) or *mad2* (n = 44), but not *Apc2* (n = 38) or *E-cad* (n = 43), significantly rescued the GSC loss phenotype associated with *Mtor* ^RNAi^. All flies were cultured for 7 days at 29°C before dissection.

In the *Drosophila* S2 and several human cell lines, Tpr/Mtor plays an important role in regulating the spindle assembly checkpoint (SAC) [26–29]. During interphase, the Tpr/Mtor-Mad1-Mad2 complex regulates generation of a premitotic anaphase inhibitor to protect genome integrity [27–29]. In Mtor-depleted *Drosophila* S2 cells, accumulation of Mad2 and Mps1 at kinetochores is significantly reduced and the cells enter anaphase prematurely [27]. Therefore, Mtor may regulate GSC maintenance and asymmetric division through SAC. To test this possibility, we expressed *UAS-Mtor*, *UAS-mad2*, *UAS-Apc2*, and *UAS-E-cad* with the *UAS-Mtor* ^RNAi^ lines using the *Nos-Gal4* driver. Expression of either *UAS-Mtor* or *UAS-mad2* significantly rescued the stem cell loss phenotypes of the *UAS-Mtor* ^RNAi^ lines (Fig 6, compared panel A to B and C), while expression of *UAS-Apc2* or *UAS-E-cad* had no function (Fig 6D, 6E and 6G). Further, we also found that expression of *UAS-mad2* significantly rescued the reduction of Apc2 localization phenotypes of the *UAS-Mtor* ^RNAi^ line (Fig 6F). Overexpression of *Mtor* (*Nos>Mtor*), *mad2* (*Nos>mad2*), *Apc2* (*Nos>Apc2*) and *E-cad* (*Nos>E-cad*) alone resulted in no abnormal phenotype (S6H–S6K Fig).

**Fig 7:**
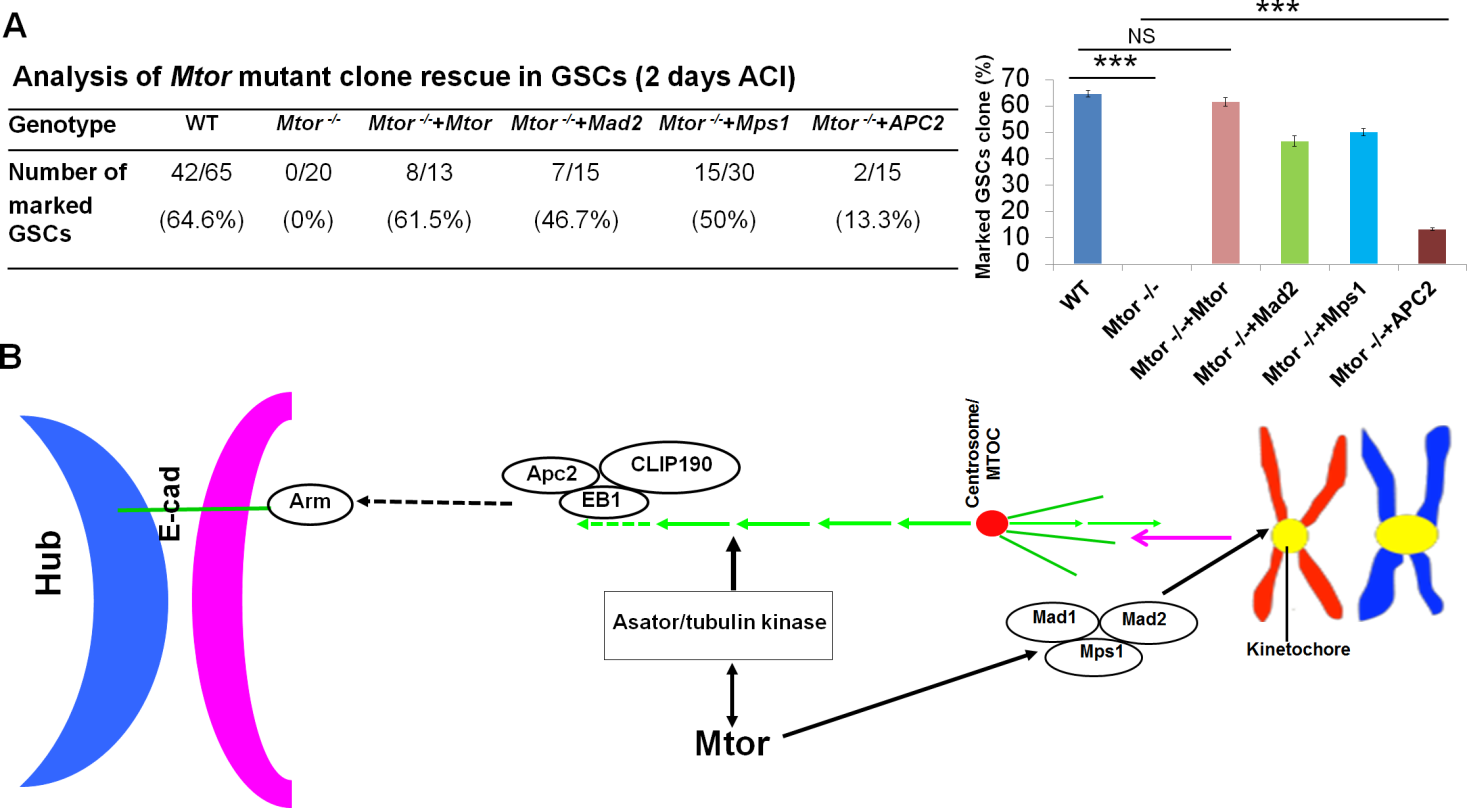
Expression of SAC components rescued the *Mtor* phenotypes in GSC clones. (A) Quantitative data of β-galactosidase–(green) negative GSC clones in testes of wild-type control (*FRT* ^42D^ *-piM*), *FRT* ^42D^ *-Mtor* ^k03905^, *FRT* ^42D^ *-Mtor* ^k03905^+*UAS-Mtor*, *FRT* ^42D^ *-Mtor* ^k03905^+*UAS-mad2*, *FRT* ^42D^ *-Mtor* ^k03905^+*UAS-mps1*, and *FRT* ^42D^ *-Mtor* ^k03905^+*UAS-Apc2* flies at 2 days ACI. *Act<CD2<Gal4/Y; FRT* ^42D^ *-Mtor* ^k03905^/ *FRT* ^42D^ *-arm-lacZ; MKRS*, *hs-flp/UAS-Mtor* (or other) flies were used to generate mosaic clones in the rescue experiments. (B) A model of how Mtor regulates asymmetric Apc2 localization, mitotic spindle formation, and asymmetric GSC division. Details are included in the text.

We further expressed *Mtor*, *mad2*, *mps1*, and *Apc2* in *Mtor-*mutant GSC mosaic clones (Fig 7A). Expression of *Mtor*, *mad2*, and *mps1* significantly rescued the GSC loss phenotypes of the *Mtor*-mutant GSC clones, while expression of *Apc2* had no significant function. These data suggest that the correct localization of Apc2 and E-cad regulated by the Mtor-SAC axis through mitotic spindle rather than the relative amounts of these proteins are important for GSC maintenance.

These above data together suggest that Mtor/Tpr regulates GSC asymmetric division and maintenance through the mitotic spindle checkpoint complex (Mps1 and Mad2).

### Expression of SAC components did not rescue CySC defects of mutant *Mtor*

The asymmetric division of CySCs occurs through a cellular mechanism strikingly distinct from the one used by GSCs. The mitotic spindle of CySCs first forms in a random location and then repositions during or near the onset of anaphase so that one pole is close to the hub cells [49]. Our above data demonstrated that depletion of *Mtor* in GSCs resulted in GSC loss, while depletion of *Mtor* in CySCs resulted in differentiation of CySCs, indicating that Mtor may function differently in GSCs and CySCs. To find out whether Mtor regulates CySCs through the SAC complex, we expressed *UAS-mad2* and *UAS-mps1* in the *UAS-Mtor* ^RNAi^ lines using the *c587-Gal4* driver. Expression of *UAS-mad2* and *UAS-mps1* did not significantly rescue the phenotypes associated with expressing *UAS-Mtor* ^RNAi^ in somatic cyst cells (they show GSC tumor phenotype; S6L and S6M Fig), suggesting that Mtor functions in GSCs and CySCs through distinct molecular pathways.

## Discussion

In male *Drosophila* GSCs, the asymmetric outcome of stem cell division is specified by an oriented spindle and cortically localized Apc2. However, the molecular mechanism that regulates asymmetric Apc2 localization and formation of the oriented spindle is unclear. In this study, we identified a nucleoporin and spindle matrix protein Tpr/Mtor that regulates GSC asymmetric division and maintenance. Loss of Mtor function results in abnormal Apc2 localization, incorrect centrosome orientation, defective mitotic spindle formation, and abnormal chromosome segregation. We further demonstrated that Mtor regulates GSC asymmetric division and maintenance through the SAC, which regulates asymmetric localization of Apc2 and E-cad. At the cortex, Apc2 and E-cad together anchor the spindles of mitotic GSCs perpendicular to the hub for asymmetric GSC division [19]. Defects in the Mtor-regulated processes may first block cytokinesis, result in polyploidy, and cause eventual loss of GSCs. We do not know how SAC/Mad2 affects APC2 localization. More experiments are needed to find the detailed molecular mechanism. However, in yeast SAC/Mad2 regulates Kar9 (the APC2 homologue) localization through the mitotic exit network (MEN) and Kip2 (kinesin-like protein) [50]. SAC/Mad2 may regulate APC2 localization through a similar mechanism in *Drosophila* male GSCs.

### Additional Mtor functions

In *Drosophila* S2 cell, Mtor forms a nuclear complex with Mad1/2 in interphase; after nuclear envelope breakdown (NEB), Mad2 is recruited to unattached kinetochores and functions in the SAC complex while Mtor reorganizes into a fusiform structure coalescent with spindle microtubules and plays a role in spindle elongation [27]. In *Mtor-*knockdown GSCs, we found that many pH3-positive GSCs exhibited lagging and scattered chromosome phenotypes, were detached from the hub; the microtubule spindles formed were incomplete, unfocused, only half, and/or without clear spindle poles. These phenotypes cannot be entirely explained by SAC defects alone. Consistent with this, expression of *Mad2* in *Mtor* GSC mosaic clones could only partially rescue the GSC loss phenotype (Fig 7A). These results suggest that there could be additional factors that together with Mad2 and Mps1 mediate Mtor’s function in GSCs. Further, expression of *Mad2* and *Mps1* did not rescue Mtor-mutant phenotypes in CySCs, suggesting that Mtor functions differentially in GSCs and CySCs. The yeast homologue of Tpr, Mlp2p, binds to the yeast spindle pole body (SPB) and promotes its efficient assembly [51]. Most recently, it has been shown that Mtor in *Drosophila* directly binds a tau-tubulin kinase, Asator, and colocalizes to the spindle region with Asator during mitosis [52]. Asator may represent a link between Mtor and the microtubule-based spindle apparatus that facilitates Mtor’s function in regulating microtubule dynamics and microtubule spindle function. Therefore, besides Mps1 and Mad2, Mtor may also regulate mitotic spindle and Apc2 localization through Asator-CLIP190-EB1 and/or the centrosome (Fig 7B).

### Mtor’s function in GSCs and CySCs

In the *Drosophila* testis, the two types of stem cells, GSCs and CySCs, use distinct molecular mechanisms for their asymmetric division. The mitotic spindle of CySCs first forms in a random location within an irregularly shaped CySC, then repositions through functional centrosomes, dynein, and the actin-membrane linker moesin during or near the onset of anaphase so that one pole is close to the hub cells [49]. Therefore, CySCs require moesin, but not Apc2, and GSCs require Apc2, but not moesin, for their orientation.

We demonstrated that expression of Mad2 and Mps1 could rescue *Mtor*-mutant phenotypes in GSCs but not in CySCs, suggesting that the Tpr/Mtor-SAC pathway regulates asymmetric Apc2 localization for asymmetric GSC division, but not asymmetric moesin localization for asymmetric CySC division. In *c587* ^*ts*^ *>Mtor* ^RNAi^ flies, the Tj-positive and Eya-positive cells were pushed away from the niche by the expanding undifferentiated germ cells, suggesting that the *Mtor*-deficient CySCs have disadvantage in niche occupancy. Consistent with the *Mtor* ^RNAi^ result, the *Mtor* mutant CySCs generated by the MARCM technique were quickly moved out of the niche and became differentiated cyst cells (Fig 3). These results together suggest that the main function of Mtor in CySCs is to regulate their attachment to the niche.

### General significance in human diseases

Dividing eukaryotic cells have to establish correct bipolar attachment of a pair of sister kinetochores residing on each mitotic chromosome to the mitotic spindle for delivering duplicated chromosomes to separate daughter cells. This process is regulated by components of the SAC. Defects in the SAC will result in improper segregation, aneuploidy, and chromosome lagging, which are linked to birth defects and cancer in animals and humans. Aneuploidy has been recognized as a major driver of cancer [53]. Our study results have demonstrated that the Tpr/Mtor through SAC regulates Apc2 localization and asymmetric GSC division. Disruption of Mtor function resulted in defective mitotic spindle and abnormal chromosome segregation. Tpr was fused together with oncogenes *met*, *raf*, and *kit* in several kinds of tumors [54–56]. It is reasonable to propose that disruption of the Tpr-SAC pathway in these tumors might lead to chromosome instability, chromosome lagging, and aneuploidy, stem cell division defects, and thereby tumor development.

## Materials and Methods

### *Drosophila* stocks and culture

Oregon R or *UAS-lacZ* ^RNAi^ was used as the wild type. *Mtor* ^k03905^ was previously described [46] and obtained from the Bloomington stock center. The P-element insertion in the *Mtor* ^k03905^ resulted in a 9-base pair duplication, including 8 base pairs of upstream genomic sequence and a duplicated +1 residue and may represent a null mutation.

To generate transgenic strains of *UAS-mad2*, *UAS-mps1*, and *UAS-Mtor*, the corresponding full-length cDNAs of the *Drosophila* genes were amplified by PCR and inserted into the *pUAST* transformation vector [57]. Second-chromosome *UAS-Apc2* transgenic flies were from David Roberts. *UAS-DEFL* (full-length *shg*) #6–3 was obtained from Kyoto stock center. *UAS-Egfr* ^DN^ was obtained from the Bloomington stock center (BL5364).

All constructs were confirmed by DNA sequencing. The UAS constructs were injected into *w* ^1118^ embryos using standard procedures.

RNAi stocks used in this study: *Mtor* ^RNAi-1^ (Vienna *Drosophila* RNAi Center-VDRC) Transformant ID 110218 [v110218]), *Mtor* ^RNAi-2^ (BL32941), *Mtor* ^RNAi-3^ (v24265), and *rl* ^RNAi^ (v35641). The sequences used for VDRC knockdown strains are available for each line at https://stockcenter.vdrc.at and sequences for Bloomington knock-down strains are available for each line at http://flystocks.bio.indiana.edu.

The following Gal4 alleles were used to drive UAS lines: *Nos-Gal4* (*nanos-Gal4VP16*) [58], obtained from the Bloomington stock center (BL4937), and *upd-Gal4* and *c587-Gal4*, provided by Ting Xie. *esg-Gal4* was obtained from Shigeo Hayashi. *Collagen (Cg)-Gal4* [37], *Dilp2-Gal4* [38], *LE-Gal4* [39], *pumpless (ppl)-Gal4* [40], *brachyenteron (byn)-Gal4* [41], *serpent* ^*hemo*^ *(srp* ^*hemo*^ *)-Gal4* [42], *Aug21-Gal4* [43], *hemolectin (hml)-Gal4* [44], and *MS1096-Gal4* [45].

Flies were raised on standard fly food at 25°C and at 65% humidity, unless otherwise indicated.

### Generating mutant GSC clones

Clones of mutant GSCs were generated as previously described [6]. To generate *Mtor*-mutant GSC clones, *FRT* ^42D^ *+* and *FRT* ^42D^ *Mtor* ^k03905^/*Cyo* virgin females were mated with males of genotype *FRT* ^42D^ *arm-lacZ*/*Cyo*; *MKRS*, *hs-flp/+*, or *FRT* ^G13^ *Mtor* ^k03905^/*Cyo* virgin females were mated with males of genotype *FRT* ^G13^ *arm-lacZ*/*Cyo*; *MKRS*, *hs-flp/+*. One- or 2-day-old adult males carrying an *arm-lacZ* transgene in *trans* to the mutant-bearing chromosome were heat shocked four times at 37°C for 1 hr, at intervals of 8–12 hr. The males were transferred to fresh food every day at 25°C. The testes were removed 1, 2, or 7 days after the first heat-shock treatment and processed for antibody staining.

### MARCM clonal analysis

To induce MARCM clones of *FRT* ^42D^ *-piM* (as a wild-type control) and *FRT* ^42D^ *-Mtor* ^k03905^, we generated the following flies: *FRT* ^42D^ *tub-Gal80/FRT* ^42D^ *Mtor* ^k03905^ (or *piM*); *MKRS*, *hs-flp/ tub-Gal4*,*UAS-mCD8*.*GFP*. Three- or 4-day-old adult male flies were heat-shocked twice at 37°C for 45 min, with an interval of 8–12 hr. The flies were transferred to fresh food daily after the final heat shock. The testes were removed at 1, 2, or 7 days after the first heat-shock treatment and processed for antibody staining.

### RNAi-mediated gene depletion

Male *UAS-RNAi* transgene flies were crossed with female virgins of genotype *Nos-Gal4*, *upd-Gal4*, *c587-Gal4; tub-Gal80* ^ts^ (*c587* ^ts^), or *esg-Gal4*, *UAS-GFP; tub-Gal80* ^ts^ (*esg* ^ts^). The flies were cultured at 18°C (Gal4 is inactive and Gal80 is active). Three- to 5-day-old adult flies with the appropriate genotype were transferred to new vials at 29°C (Gal4 is active and Gal80 is inactive) for 3 or 7 days before dissection.

### Immunofluorescence staining and microscopy

Normal immunofluorescence staining was performed as described previously with some modifications [6]. Briefly, testes were dissected in phosphate-buffered saline (PBS), transferred to 4% formaldehyde in PBS, and fixed for 30 minutes. The testes were then washed in PBST (PBS containing 0.1% Triton X-100) for 3 times, 10 Minutes each time, then blocked with 5% goat serum in PBST for 1 hour. Samples were the incubated with primary antibody in PBST at 4°C overnight. Samples were washed for 30 minutes (three 10-minute washes) in PBST, incubated with secondary antibody in PBST at room temperature for 2 hours, washed as above, and mounted in VECTASHIELD with DAPI (Vector Labs).

For the γ-tubulin staining, testes were dissected in PBS, transferred to 4% formaldehyde in PBS, and fixed for 20 minutes, followed by incubation with methanol for 10 minutes. Then washed for 10 minutes with PBST, and two 10-minutes washes with 5% goat serum PBST. Then incubated in primary antibody in 5% goat serum in PBST overnight at 4°C. Then washed three times, 15 min each in PBST, followed with 2 hrs incubation with secondary antibody in 5% goat serum in PBST. Then washed for at least an hour with PBST, and mounted as above.

Caspase-3 activity was assessed using Live Green Caspase Detection Kits (I35106, Molecular Probes) according to standard protocol.

Confocal images were obtained by using a Zeiss LSM510 system, and were processed with Adobe Photoshop 7.0. GSCs were scored as Vasa-positive cells adjacent to the hub (detected using Fas3) and containing dot spectrosome (detected using 1B1). Only image with a clear view of the complete hub were used.

The following antisera were used: rabbit polyclonal anti-Vasa antibody (1:5000; gift from R. Lehmann), rabbit polyclonal anti-β-Gal antibody (1:1000; Cappel), mouse monoclonal anti-β-Gal antibody (1:100; Invitrogen), mouse monoclonal anti-Hts antibody 1B1 (1:4; Developmental Studies Hybridoma Bank [DSHB]), mouse monoclonal anti-Fas 3 antibody (1:10; DSHB), rat polyclonal anti-Tj (1:400; Li et al. [59], mouse monoclonal anti-Eya (1:20, DSHB), mouse monoclonal anti-Dl (1:20; DSHB), mouse monoclonal anti-Pros (1:50; DSHB), rabbit polyclonal anti-GFP antibody (1:200; Molecular Probes), mouse monoclonal anti-GFP antibody (1:100; Invitrogen), rabbit polyclonal anti-Caspase 3 antibody (1:1000; gift from B. Hay); rabbit polyclonal anti-Thr3-phosphorylated histone H3 antibody (1:200; upstate), mouse monoclonal anti-α-tubulin antibody (1:100; Sigma), mouse monoclonal anti-γ-tubulin antibody (1:100; Sigma), guinea pig polyclonal anti-Zfh1 (1:2000; gift from J. Skeath), mouse monoclonal anti-Mtor antibody (1:100; gift from K. Johansen), rabbit polyclonal anti-Apc2 antibody (1:5000; gift from M. Bienz). Secondary antibodies were goat anti-mouse, goat anti-guinea pig, and goat anti-rabbit IgG conjugated to Alexa 488 or Alexa 568 (1:400; Molecular Probes). DAPI (Molecular Probes) was used to stain DNA.

### Score of centrosome and spindle orientation

We scored the centrosome misorientation and spindle misorientation following the protocol described by Yamashita et al. [19, 60]. Specifically, centrosome misorientation was noted when neither of two centrosomes were closely associated with hub-GSC interface during interphase and at mitosis. Spindle misorientation was scored when neither of the two spindle poles was closely associated with hub-GSC interface during mitosis [19, 60].

### Statistical analyses

Statistical analyses were performed using Microsoft Excel 2010 or GraphPad Prism 6 software. Data are shown as means ± SD or standard error of the mean (SEM). P-values were obtained between two groups using the Student’s t-test or between more than two groups by analysis of variance (ANOVA).

## Supporting Information

S1 Fig Mtor’s function is required in CySCs. (A and B) GSCs in testes of *c587* ^ts^ *>LacZ* ^RNAi^ (A), *c587* ^ts^ *>Mtor* ^RNAi-2^ (B), *c587* ^ts^ *>Egfr* ^DN^ (C), and *c587* ^ts^ *>rl* ^RNAi^ (D) flies were examined by staining with anti-vasa (red, marks all germ cells including GSCs), Fas3 (green, hub cells), anti-1B1 (green in dot and branched marks the spectrosomes and fusomes respectively) and DAPI (blue). Hub cells are marked by asterisks. In A, white dotted circle with white arrow marks GSCs and yellow dotted circle with yellow arrow marks GB. In B-D, white dotted circles mark expended GSCs or GSC-like cells and white arrowheads point to 1B1 dots. (E-G) GSCs in testes of *c587* ^ts^ *>LacZ* ^RNAi^ (E, G) and *c587* ^ts^ *>Mtor* ^RNAi-2^ (F, H). GSCs in testes of *c587* ^ts^ *>LacZ* ^RNAi^ (E) and *c587* ^ts^ *>Mtor* ^RNAi-2^ (F) flies were examined by staining with anti-vasa (red, marks all germ cells including GSCs), Fas3 (green, hub cells), anti-Eya (late stage cyst cells, green arrows) and DAPI (blue). GSCs in testes of *c587* ^ts^ *>LacZ* ^RNAi^ (G) and *c587* ^ts^ *>Mtor* ^RNAi-2^ (H) flies were examined by staining with anti-vasa (green, marks all germ cells including GSCs), Tj (red, CySC and early cyst cells), and DAPI (blue). In E anf G, white dotted circle with white arrowhead marks GSCs and yellow dotted circle with yellow arrow marks GB. In F and H, white dotted circles mark expended GSCs or GSC-like cells and red arrows point to Tj-positive cells. Green arrows in E and F point to Eya-positive cells. Scale bars are 10 μm in all panels. All flies were cultured for 7 days at 29°C before dissection.
(TIF)

S2 Fig Mtor’s function is not required in hub cells, ISCs, and several other cell types. (A and B) GSCs in testes of *upd>LacZ* ^RNAi^ (A) and *upd>Mtor* ^RNAi-2^ (B) flies were examined by staining with anti-vasa (red, marks all germ cells including GSCs (white arrowhead and white dotted circle), anti-1B1 (green in dot and branched marks the spectrosomes and fusomes respectively), and DAPI (blue). (C and D) ISCs in adult posterior midguts of wild-type control (*esg* ^ts^ *>lacZ* ^RNAi^) (C) or *esg* ^ts^ *>Mtor* ^RNAi-2^ (D) flies were examined by staining with anti-GFP (green, marks ISCs and enteroblast cells), anti-delta (red, ISCs), anti-Pros (red nuclear staining marks enteroendocrine cells) and DAPI (blue). Scale bars are 10 μm in all panels. All flies were cultured for 7 days at 29°C before dissection. (E) *UAS-Mtor* ^RNAi-2^ was expressed with the nine Gal4s listed. Only the *ms1096>Mtor* ^RNAi-2^ flies had wing phenotypes, and the other eight Gal4*>Mtor* ^RNAi-2^ flies had no abnormal phenotypes. All flies were cultured for 7 days at 29°C before examination.
(TIF)

S3 Fig Mtor is required for GSC maintenance. (A-B) GSCs in testes of wild-type control (*Nos>lacZ* ^RNAi^) (A) or *Nos>Mtor* ^RNAi-2^ (B), were examined by staining with the indicated antibodies. CySCs (Zfh1 positive cells) located outside GSCs in wild type testis (A), however some CySCs positioned adjacent to the hub cells after some GSCs were depleted in Mtor-deficient testis (B). (C-D’) Testes of wild-type control (*Act* ^ts^ *>lacZ* ^RNAi^) (C-C’) or *Act* ^ts^ *>Mtor* ^RNAi-2^ (D-D’) flies were stained with anti-Mtor (green) and DAPI (blue). Testes of wild-type control (E,F) flies were stained with anti-Mtor (green), NUP98 (red), and DAPI (blue). The broken line circles with asterisks mark the hubs and other broken line circles mark GSCs. All flies were cultured for 7 days at 29°C before dissection. Scale bars are 10 μm in all panels.
(TIF)

S4 Fig The GSC loss in Mtor-deficient testes is not through apoptosis. (A,B) Testes of wild-type control (*Nos>lacZ* ^RNAi^) (A), and a testis with *FRT* ^42D^ *-Mtor* ^k03905^ mosaic clones (1 day ACI, B) were examined by staining with Caspase 3 (Cas3, green) and DAPI (blue). (C,D) Expression of *p35* in the testis of *Nos>Mtor* ^RNAi-2^ (C) or in the testis with *FRT* ^42D^ *-Mtor* ^k03905^ mosaic clones (2 days ACI, D) did not rescue the GSC loss phenotypes of the *Mtor* mutants. We assessed the rescue of GSC death in the testis of *Nos>Mtor* ^RNAi-2^ (C) or in the testis with *FRT* ^42D^ *-Mtor* ^k03905^ (D) by counting the number of GSC attached to the hub in C and number of GSC clones in D. The testes were examined by staining with Fas3, LacZ (green), Vasa (red), and DAPI (green). White circle lines indicate GSC clones. Asterisks mark hub cells. (E,F) Apc2 (white) localization was examined in testes of *Nos>Mtor* ^RNAi-2^ flies. White arrowheads point to Apc2 localization to the hub-GSC interface and red arrows point to Apc2 localization over the GSC cortex. The yellow stars indicate the hub. Scale bars are 10 μm in all panels.
(TIF)

S5 Fig Mtor function is required for the correct centrosome orientation, mitotic spindle formation, and chromosome segregation. (A-C) Centrosome orientation was examined in wild-type control (*Nos>lacZ* ^RNAi^) (A) and *Nos>Mtor* ^RNAi-2^ (B, C) testes. The testes were stained with γ-tubulin (green) and pH3 (red) and DAPI (blue). The broken-line circles with asterisks indicate the hubs. GSCs are highlighted with yellow dotted lines. White arrowheads point to the centrosome localization. Yellow arrows in B and C point to abnormally condensed chromosomes. (D) Abnormal chromosome segregation was observed in *Nos>Mtor* ^RNAi-2^ GSCs. The testes were stained with the α-tubulin (green) and pH3 (red) and DAPI (blue). The broken-line circle with asterisk marks the hub. Yellow arrows point to abnormally segregated chromosomes and white arrows point to abnormal mitotic spindles. (E-H) Mitotic spindles and chromosomes were examined in testes of wild-type control (*Nos>lacZ* ^RNAi^) (E) and *Nos>Mtor* ^RNAi-2^ (F-H) flies. The testes were stained with the α-tubulin (green), γ-tubulin (red), pH3 (red) and DAPI (blue). The white broken-line circles with asterisks mark the hubs. White arrowheads in E point to the centrosome localization. Yellow arrow in F points to the lagging chromosome. Yellow arrows in G and H point to abnormally condensed and segregated chromosomes. All flies were cultured for 7 days at 29°C before dissection. Scale bars are 10 μm in all panels.
(TIF)

S6 Fig Mtor function is required for the correct centrosome orientation, mitotic spindle formation, and chromosome segregation. (A-D) Mitotic spindles and chromosomes were examined in testes of Control (*Nos>lacZ* ^RNAi^) (A), *Nos>Mtor* ^RNAi-3^ (B,D) and *Nos>Mtor* ^RNAi-2^ (C,E) flies. The testes were stained with the pH3 (red), α-tubulin (green) and DAPI (blue). The white broken-line circles with asterisks mark the hubs. The yellow broken-line circles mark GSCs. White arrow in B points to the spindle bridge of segregated chromosomes. Yellow arrows in C and D point to abnormally condensed and segregated chromosomes. Yellow arrow in E points to the lagging chromosome. (F,G) mitotic spindles and chromosomes were examined in adult posterior midgut ISCs of *esg>Mtor* ^RNAi-2^ and stain with pH3 (red), α-tubulin (green) and DAPI (blue). (H-K) GSCs in testes of *Nos>Mtor* (H), *Nos>mad2* (I), *Nos>Apc2* (J), and *Nos>E-cad* (K) flies were examined by staining with the anti-vasa (red, marks all germ cells including GSCs), Fas3 (green, hub cells), anti-1B1 (green in dot and branched marks the spectrosomes and fusomes respectively) and DAPI (blue). White dotted circles with white arrow mark GSCs, yellow dotted circles with yellow arrow mark GB, red dotted circles mark CySCs, and asterisks mark hubs. GSCs in testes of (L) *c587>Mtor* ^*RNAi-2*^ *+UAS-Mad2* and (M) *c587>Mtor* ^*RNAi-2*^ *+UAS-Mps1* flies were examined by staining with the anti-vasa (red, marks all germ cells including GSCs), Fas3 (green, hub cells), anti-1B1 (green in dot and branched marks the spectrosomes and fusomes respectively) and DAPI (blue). Dotted lines mark GSC tumor phenotype, and asterisks mark hub cells. Scale bars are 10 μm in all panels. All flies were cultured for 7 days at 29°C before dissection.
(TIFF)
