# Supplementary figures and images for: The Nuclear Matrix Protein Megator Regulates Stem Cell Asymmetric Division through the Mitotic Checkpoint Complex in Drosophila Testes
Source: PLoS Genet. 2015 Dec 29;11(12):e1005750. doi: 10.1371/journal.pgen.1005750 (PMC4703072; doi:10.1371/journal.pgen.1005750)

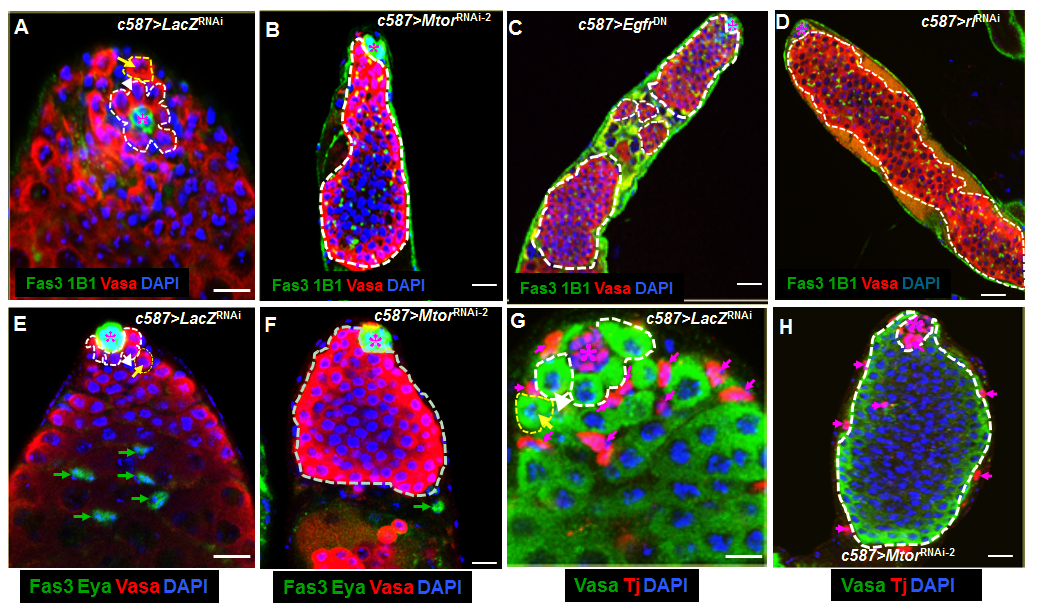

Supplement: S1 Fig — (A and B) GSCs in testes of c587 ts >LacZ RNAi (A), c587 ts >Mtor RNAi-2 (B), c587 ts >Egfr DN (C), and c587 ts >rl RNAi (D) flies were examined by staining with anti-vasa (red, marks all germ cells including GSCs), Fas3 (green, hub cells), anti-1B1 (green in dot and branched marks the spectrosomes and fusomes respectively) and DAPI (blue). Hub cells are marked by asterisks. In A, white dotted circle with white arrow marks GSCs and yellow dotted circle with yellow arrow marks GB. In B-D, white dotted circles mark expended GSCs or GSC-like cells and white arrowheads point to 1B1 dots. (E-G) GSCs in testes of c587 ts >LacZ RNAi (E, G) and c587 ts >Mtor RNAi-2 (F, H). GSCs in testes of c587 ts >LacZ RNAi (E) and c587 ts >Mtor RNAi-2 (F) flies were examined by staining with anti-vasa (red, marks all germ cells including GSCs), Fas3 (green, hub cells), anti-Eya (late stage cyst cells, green arrows) and DAPI (blue). GSCs in testes of c587 ts >LacZ RNAi (G) and c587 ts >Mtor RNAi-2 (H) flies were examined by staining with anti-vasa (green, marks all germ cells including GSCs), Tj (red, CySC and early cyst cells), and DAPI (blue). In E anf G, white dotted circle with white arrowhead marks GSCs and yellow dotted circle with yellow arrow marks GB. In F and H, white dotted circles mark expended GSCs or GSC-like cells and red arrows point to Tj-positive cells. Green arrows in E and F point to Eya-positive cells. Scale bars are 10 μm in all panels. All flies were cultured for 7 days at 29°C before dissection. (TIF) [file pgen.1005750.s001.tif]

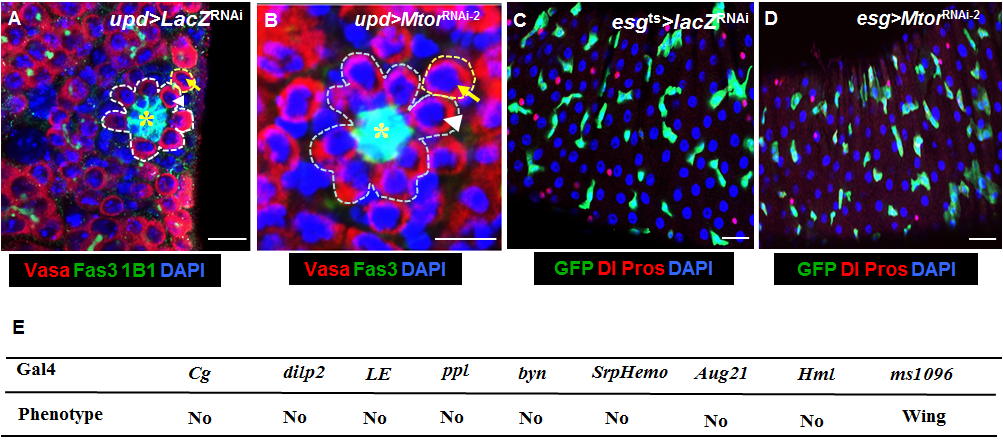

Supplement: S2 Fig — (A and B) GSCs in testes of upd>LacZ RNAi (A) and upd>Mtor RNAi-2 (B) flies were examined by staining with anti-vasa (red, marks all germ cells including GSCs (white arrowhead and white dotted circle), anti-1B1 (green in dot and branched marks the spectrosomes and fusomes respectively), and DAPI (blue). (C and D) ISCs in adult posterior midguts of wild-type control (esg ts >lacZ RNAi) (C) or esg ts >Mtor RNAi-2 (D) flies were examined by staining with anti-GFP (green, marks ISCs and enteroblast cells), anti-delta (red, ISCs), anti-Pros (red nuclear staining marks enteroendocrine cells) and DAPI (blue). Scale bars are 10 μm in all panels. All flies were cultured for 7 days at 29°C before dissection. (E) UAS-Mtor RNAi-2 was expressed with the nine Gal4s listed. Only the ms1096>Mtor RNAi-2 flies had wing phenotypes, and the other eight Gal4>Mtor RNAi-2 flies had no abnormal phenotypes. All flies were cultured for 7 days at 29°C before examination. (TIF) [file pgen.1005750.s002.tif]

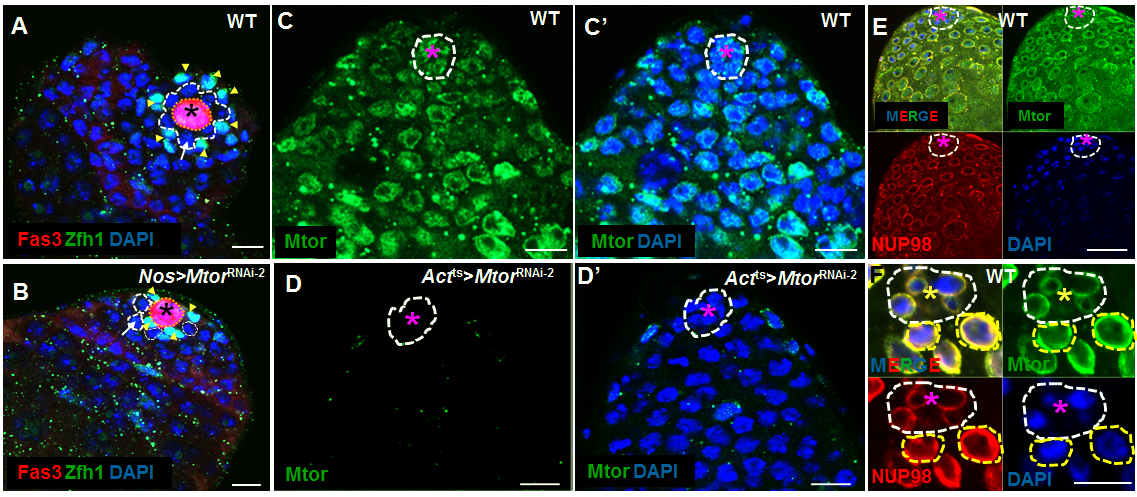

Supplement: S3 Fig — (A-B) GSCs in testes of wild-type control (Nos>lacZ RNAi) (A) or Nos>Mtor RNAi-2 (B), were examined by staining with the indicated antibodies. CySCs (Zfh1 positive cells) located outside GSCs in wild type testis (A), however some CySCs positioned adjacent to the hub cells after some GSCs were depleted in Mtor-deficient testis (B). (C-D’) Testes of wild-type control (Act ts >lacZ RNAi) (C-C’) or Act ts >Mtor RNAi-2 (D-D’) flies were stained with anti-Mtor (green) and DAPI (blue). Testes of wild-type control (E,F) flies were stained with anti-Mtor (green), NUP98 (red), and DAPI (blue). The broken line circles with asterisks mark the hubs and other broken line circles mark GSCs. All flies were cultured for 7 days at 29°C before dissection. Scale bars are 10 μm in all panels. (TIF) [file pgen.1005750.s003.tif]

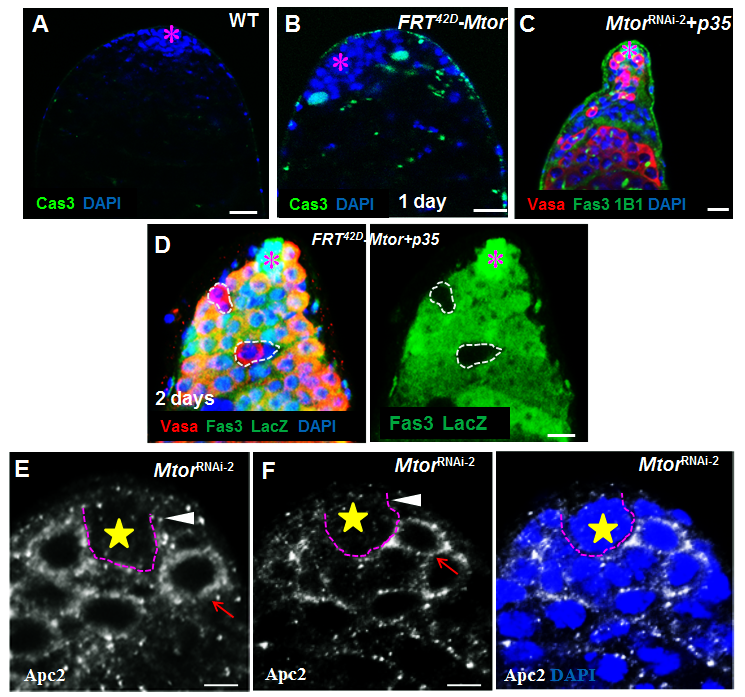

Supplement: S4 Fig — (A,B) Testes of wild-type control (Nos>lacZ RNAi) (A), and a testis with FRT 42D -Mtor k03905 mosaic clones (1 day ACI, B) were examined by staining with Caspase 3 (Cas3, green) and DAPI (blue). (C,D) Expression of p35 in the testis of Nos>Mtor RNAi-2 (C) or in the testis with FRT 42D -Mtor k03905 mosaic clones (2 days ACI, D) did not rescue the GSC loss phenotypes of the Mtor mutants. We assessed the rescue of GSC death in the testis of Nos>Mtor RNAi-2 (C) or in the testis with FRT 42D -Mtor k03905 (D) by counting the number of GSC attached to the hub in C and number of GSC clones in D. The testes were examined by staining with Fas3, LacZ (green), Vasa (red), and DAPI (green). White circle lines indicate GSC clones. Asterisks mark hub cells. (E,F) Apc2 (white) localization was examined in testes of Nos>Mtor RNAi-2 flies. White arrowheads point to Apc2 localization to the hub-GSC interface and red arrows point to Apc2 localization over the GSC cortex. The yellow stars indicate the hub. Scale bars are 10 μm in all panels. (TIF) [file pgen.1005750.s004.tif]

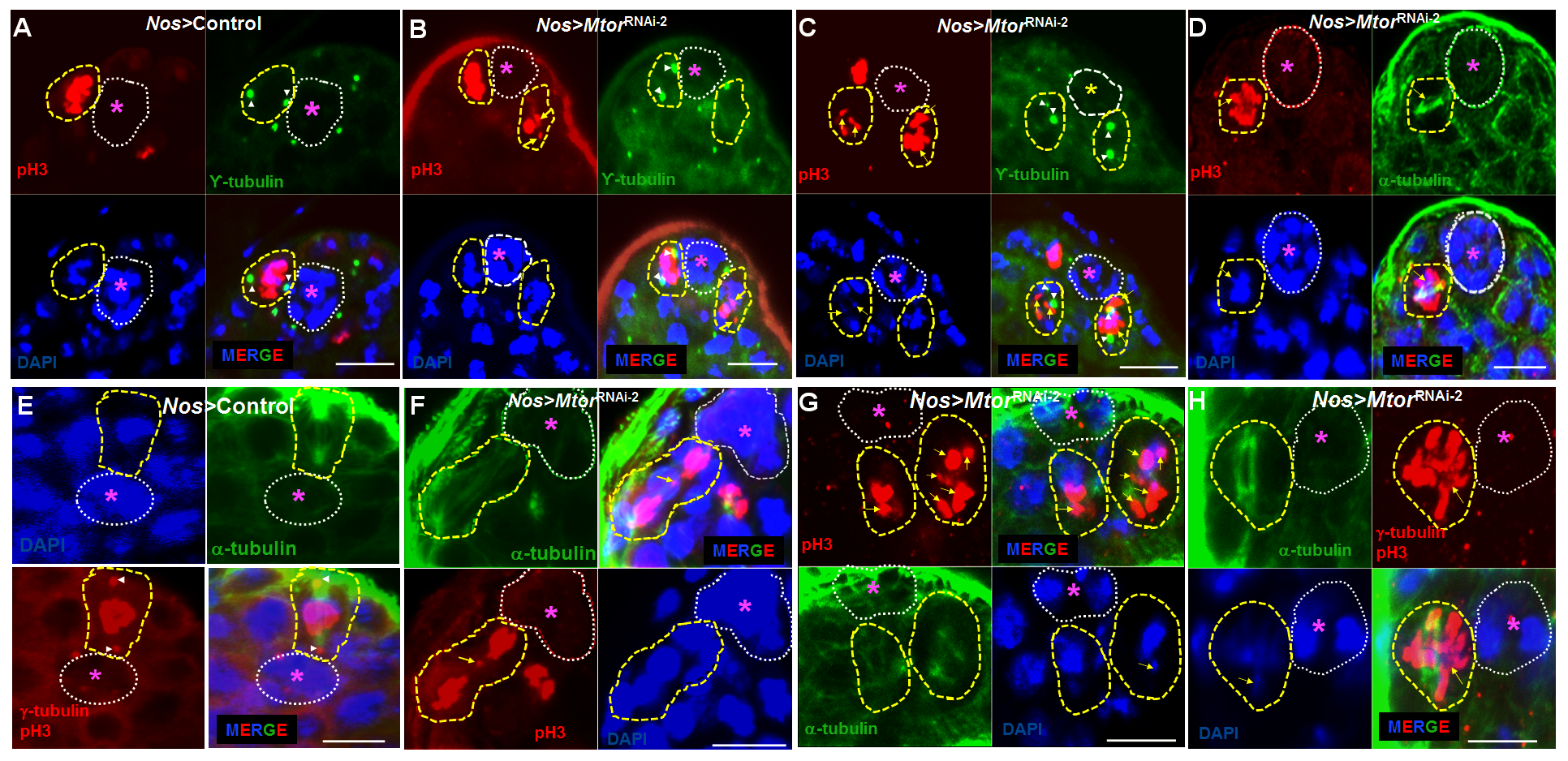

Supplement: S5 Fig — (A-C) Centrosome orientation was examined in wild-type control (Nos>lacZ RNAi) (A) and Nos>Mtor RNAi-2 (B, C) testes. The testes were stained with γ-tubulin (green) and pH3 (red) and DAPI (blue). The broken-line circles with asterisks indicate the hubs. GSCs are highlighted with yellow dotted lines. White arrowheads point to the centrosome localization. Yellow arrows in B and C point to abnormally condensed chromosomes. (D) Abnormal chromosome segregation was observed in Nos>Mtor RNAi-2 GSCs. The testes were stained with the α-tubulin (green) and pH3 (red) and DAPI (blue). The broken-line circle with asterisk marks the hub. Yellow arrows point to abnormally segregated chromosomes and white arrows point to abnormal mitotic spindles. (E-H) Mitotic spindles and chromosomes were examined in testes of wild-type control (Nos>lacZ RNAi) (E) and Nos>Mtor RNAi-2 (F-H) flies. The testes were stained with the α-tubulin (green), γ-tubulin (red), pH3 (red) and DAPI (blue). The white broken-line circles with asterisks mark the hubs. White arrowheads in E point to the centrosome localization. Yellow arrow in F points to the lagging chromosome. Yellow arrows in G and H point to abnormally condensed and segregated chromosomes. All flies were cultured for 7 days at 29°C before dissection. Scale bars are 10 μm in all panels. (TIF) [file pgen.1005750.s005.tif]

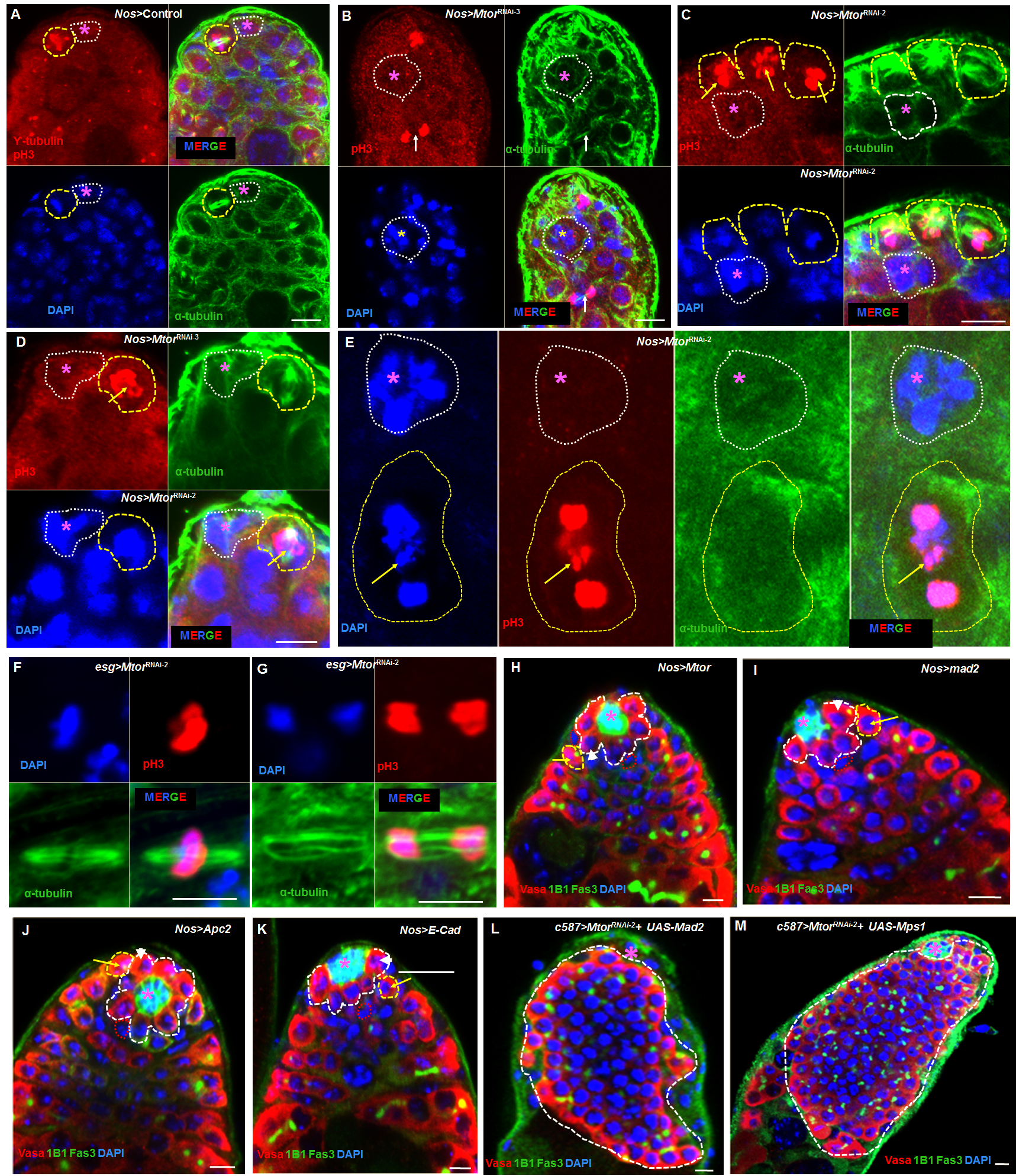

Supplement: S6 Fig — (A-D) Mitotic spindles and chromosomes were examined in testes of Control (Nos>lacZ RNAi) (A), Nos>Mtor RNAi-3 (B,D) and Nos>Mtor RNAi-2 (C,E) flies. The testes were stained with the pH3 (red), α-tubulin (green) and DAPI (blue). The white broken-line circles with asterisks mark the hubs. The yellow broken-line circles mark GSCs. White arrow in B points to the spindle bridge of segregated chromosomes. Yellow arrows in C and D point to abnormally condensed and segregated chromosomes. Yellow arrow in E points to the lagging chromosome. (F,G) mitotic spindles and chromosomes were examined in adult posterior midgut ISCs of esg>Mtor RNAi-2 and stain with pH3 (red), α-tubulin (green) and DAPI (blue). (H-K) GSCs in testes of Nos>Mtor (H), Nos>mad2 (I), Nos>Apc2 (J), and Nos>E-cad (K) flies were examined by staining with the anti-vasa (red, marks all germ cells including GSCs), Fas3 (green, hub cells), anti-1B1 (green in dot and branched marks the spectrosomes and fusomes respectively) and DAPI (blue). White dotted circles with white arrow mark GSCs, yellow dotted circles with yellow arrow mark GB, red dotted circles mark CySCs, and asterisks mark hubs. GSCs in testes of (L) c587>Mtor RNAi-2 +UAS-Mad2 and (M) c587>Mtor RNAi-2 +UAS-Mps1 flies were examined by staining with the anti-vasa (red, marks all germ cells including GSCs), Fas3 (green, hub cells), anti-1B1 (green in dot and branched marks the spectrosomes and fusomes respectively) and DAPI (blue). Dotted lines mark GSC tumor phenotype, and asterisks mark hub cells. Scale bars are 10 μm in all panels. All flies were cultured for 7 days at 29°C before dissection. (TIFF) [file pgen.1005750.s006.tiff]
